# Endoscopic transcanal myringoplasty with anterior tab flap underlay technique: An analysis of 35 cases

**DOI:** 10.1016/j.amsu.2022.104135

**Published:** 2022-07-11

**Authors:** Thanh Thai Le, Doan Minh Nhat Vo, Thi My Duong, Nguyen Nguyen

**Affiliations:** Department of Otolaryngology, Hue University of Medicine and Pharmacy, Hue University, 06 Ngo Quyen Street, Vinh Ninh Ward, Hue City, Thua Thien Hue province, Viet Nam

**Keywords:** Endoscopic transcanal, Myringoplasty, Anterior tab flap, Anterior perforation, Underlay technique

## Abstract

**Introduction:**

The repair of the tympanic membrane can present a problem, especially in anterior perforation, because the anterior portion was not enough to inadequate contact between tympanic membrane remnant and graft. Various surgical techniques were recommended to achieve an acceptable graft success rate in anterior perforation. Endoscopic transcanal myringoplasty with anterior tab flap could provide the better stability of the graft.

**Objective:**

The aim of this study was to report the minimally invasive technique for the anterior tympanic membrane perforation closure and investigate the graft success rate of this technique.

**Patients and methods:**

We performed a prospective, randomized study of 35 patients who consulted the otorhinolaryngology department at the university hospital for surgery of perforation tympanic membrane repair.

**Results:**

The average age was 35.1 ± 11.9 years. The size of the perforation was dominant at small-size and large-size, 51.4%, 34.3%, respectively. There was a significant difference between the Preoperative air conduction of small and large perforations (34.44 8.68 and 49.79 14.54, respectively). Of 35 patients, 31 (88.6%) had closure of their perforations. The mean preoperative ABG was 24.11 ± 10.79 dB, while The mean postoperative ABG was 13.97 ± 10.03 dB (p < 0.05). Approximately 34.3% patients had ABG within 20 dB preoperatively, which increased to 82.9% after intervention (p < 0.05).

**Conclusions:**

The endoscopic transcanal myringoplasty with anterior tab flap underlay technique is a safe, suitable and effective method for cases with anterior tympanic membrane perforations, and showed improvement in postoperative hearing.

## Highlights

• The repair of the anterior tympanic membrane perforation can present a problem because of the unstability of the graft.
• Endoscopic transcanal myringoplasty with anterior tab flap is a suitable alternative technique.
• The graft success rate was 88.6% in our study.
• The percentage of air bone gap <20 dB increases from 34.3% (before surgery) to 82.9% (after surgery).

## 1 Introduction

Myringoplasty known as tympanoplasty type I, introduced a long time ago, used to repair tympanic membrane perforations in chronic otitis media or unhealed traumatic eardrum perforations. The aim of the surgery is to reconstruct the anatomic tympanic membrane to prevent infection and improve hearing function for patients [10]. There is a wide range of techniques of myringoplasty that were described and the two most common techniques are the underlay and the overlay techniques [8,9]. The underlay technique is easier to perform, less time-consuming and suitable for posterior perforations, thus, it is often preferred by otologists [7]. However, for anterior tympanic membrane perforations, especially close to the anterior annulus, the underlay technique is difficult to repair because of the anterior bony overhang and lack of vascularity and the graft may fall to the middle ear cavity [12]. This is always a challenge for surgeons. According to a recent study by Sajid T (2017), anterior perforations were found to fail in 41,7% of the cases, while central perforation failed in only 5,4% of the cases; the intact graft success rate of the marginal perforation was significantly decreased, only 47,1% while it was 94,9% in cases where annulus was not involved [11]. Therefore, ear surgeons have constantly tried to improve surgical techniques to apply to these cases.

One of the innovative surgical methods for this case is pulling a small tab of the graft through a small tunnel under the anterior annulus, called the “anterior hitch method”, introduced by Primorse and Kerr in 1986. Then many otologists applied techniques with the same approach but in various names and showed that the graft success rate is increased compared with standard underlay [6].

In Vietnam, this technique has not been popular. Therefore, this study aimed to apply the underlay myringoplasty with anterior tab flap for the anterior tympanic membrane perforations close to the anterior margin and estimate the efficacy of this technique.

## 2 Material and methods

35 patients with an anterior quadrant tympanic membrane perforation were enrolled in this study and they underwent the procedure at the medical institution between October 2019 and December 2020. All patients completed the minimum three-month follow-up period. The patients with prior otologic surgery, ossicular chain disease, cholesteatoma and retraction pockets were excluded in this study. All procedures conducted in this study were by the 1964 Helsinki Declaration. The study protocol was approved by our corresponding Institutional Review Board (IRB) before starting the study. Informed consent was obtained from all the patients preoperatively. The work has been reported in line with the STROCSS [17]. A measuring template with imprinting the graph grid 1 mm × 1 mm square was made from the thin and transparent plastic paper. These templates were used to measure the size of the TM perforation and they were sterilized in cidex, before measuring the perforation. The sizes of perforation were intraoperatively measured and they were categorized in comparison with the average surface area of the TM (64.3 mm^2^). The sizes of TM perforation have been categorized as follows:

1–32 mm^2^, <50% of the average surface area of the TM involved by perforation
>32–48 mm^2^, 50%–75% of the average surface area of the TM
>48 mm^2^, >75% of the average surface area of the TM

The preoperative and postoperative three-month pure-tone audiometry (PTA) were performed at the following frequencies: 250, 500, 1000, 2000, 3000, 4000, 8000 Hz. Pure-tone averages of air and bone conduction were calculated at the frequencies of 500, 1000, 2000, 3000 Hz. Then, average air-bone gaps were computed at the same frequencies. The closure of the TM after 3 months has been considered successful.

### 2.1 Surgical technique

The same surgeon operated the patients under general anesthesia. The standard transcanal approach was used with a 0° rigid endoscope. Using an angled pick, the edges of the TM perforation were freshened to remove all squamous epithelium that unfolded into the perforation. Also, the refreshing of these edges will promote migration of epithelium and then successful closure of the perforation. We used temporalis fascia graft for grafting and it was harvested through a small incision # 1 cm just above the ear. All muscle and adipose tissue were resected off the graft, then the graft was crushed and dried. Finally, the graft was trimmed off and a triangle shape tag was created at the anterior part of the graft. The incisions were created at the anterior and posterior parts of the external auditory canal. The posterior tympanomeatal flap was performed and the mallear area of TM was protected. A 2 mm incision of the anterior part was made as far as 2 mm from lateral to the annulus. Using the Rosen needle, we created an anterior meatal tunnel with careful elevation of the anterior canal skin and fibrous annulus. After the posterior tympanomeatal flap was reflected anteriorly, the graft was positioned medially to the TM remnant and it was tucked underneath the malleus handle and over the incus. Anterior tab of the graft was then hooked into the tunnel (underneath the anterior flap and the annulus) with the right-angle hook. This procedure would tightly anchor the fascia graft anteriorly. Then, the middle ear cavity was filled with absorbable gelatin sponges. The posterior and anterior tympanomeatal flaps were repositioned and the annulus was carefully replaced in the sulcus. The levofloxacin-soaked sponges were placed over the TM. A 2 cm medical gauze pack impregnated with 5% povidone-iodine solution was introduced into the canal and it was removed on postoperative day 3. Otoscopic assessment was performed pre-operation, and then 1 week, 1 month, 3 month post-operation by a 0° endoscope.

### 2.2 Statistical analysis

The Statistical Packages for Social Sciences (SPSS) version 22 (Armonk, NY: IBM Corp) was used to analyze the collected data. The analysis of normality was identified by the Shapiro-Wilk test. The differences in audiometric findings pre- and postoperation were evaluated by using the Wilcoxon test. The Kruskal-Wallis Test was used with a 0,05 significance level to determine the relation between the preoperative air conduction and these groups. All study protocols were approved by our research ethics committee (decision number H2018/031) on May 10, 2018.

## 3 Results

Thirty-five patients were enrolled in the study. The average age of the participants was 35.1 ± 11.9, with the youngest patient was 19 years old and the oldest being 64. Of all, 34.3% were men and 65.7% were women. As to the perforation side of the TM, 60% (21 patients) had it on the left ear and 40% (14 patients) on the right side. According to the size of perforations, 51.4% of the patients had a small-size perforation (<25%), 34.3% of the patients had big perforation (>50%), whereas only 5% had medium-sized perforation (25%–50%) (Table 1). The mean time consumption was 55.71 min, and there were no cases of complications such as anterior blunting or lateralization of the graft.

**Table 1:** Participants’ characteristics.

Variables		Total (n = 35)
		Frequency (%)
Age	$\bar{X}$± SD	35.14 ± 11.90
	Min - Max	19–64
Sex, n (%)	Male	12 (34.3)
	Female	23 (65.7)
Affected ear, n (%)	Right	14 (40.0)
	Left	21 (60.0)
Size of perforations, n (%)	≤ 25%	18 (51.4)
	> 25–50%	5 (14.3)
	> 50%	12 (34.3)

Preoperative air conduction was found to be mean SD (34.44 8.68) in small perforations (25%); in medium perforations (25–50%), mean SD (33.5 5.96); in large perforations, mean SD (49.79 14.54). We conduct a Kruskal-Wallis Test using a 0.05 significance level to determine if there is a statistically significant difference between the Preoperative air conduction across these three groups. The p-value of the test is less than 0.05. Then we continue to make pairwise comparisons, and the result shows that there is a statistically significant difference between the Preoperative air conduction of small perforations (25%) and large perforations (50%). The size of the perforation was a significant factor that affected the hearing loss level. It indicated that the larger the size of perforation was, the more serious the hearing loss was (Table 2).

**Table 2:** Relation between preoperative air conduction and size of perforation.

Size of perforations	Preoperative air conduction (mean ± SD)	p
≤ 25%	34.44 ± 8.68	p < 0.05
> 25–50%	33.5 ± 5.96	
> 50%	49.79 ± 14.54	
Total	39.57 ± 12.90	

**Fig. 1:**
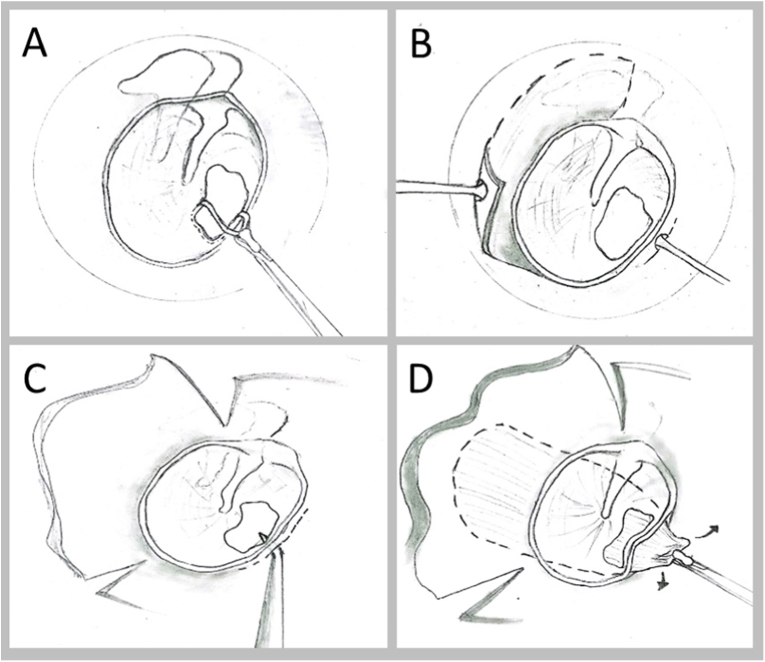
Underlay myringoplasty with anterior tab flap A. A rim of tissue is removed from the edges of an anterior perforation. B. Incision just lateral to the anterior portion of annulus. C. The canal skin is elevated creating a tunnel anteriorly. D. The anterior tab is pulled through the anterior canal skin tunnel underneath the anterior portion of the annulus.

Surgical success was defined as an intact graft after three month. The distribution of overall results is summarized in Table 3. At 3 months of follow-up, the overall success rate was 88.6%. There are 4 patients (11.4%) who had reperforation but smaller (Table 3). The mean SD of postoperative air conduction established after myringoplasty was 28.75 ± 12.82. There was a significant decrease in the mean air conduction (10.82 ± 5.99 dB) from the preoperation to the postoperation (p < 0.05). This result showed an average improvement in hearing (Table 4) (see Fig. 1).

**Table 3:** The graft success rate (GSR).

	n	%
Healed	31	88.6
Slit	4	11.4
Total	35	100.0

**Table 4:** Preoperative and Postoperative air conduction.

	$\bar{X}$± SD	Differences	p
Pre-operative	39.57 ± 12.90	10.82 ± 5.99	p < 0.05
Post-operative	28.75 ± 12.82		

**Fig. 2:**
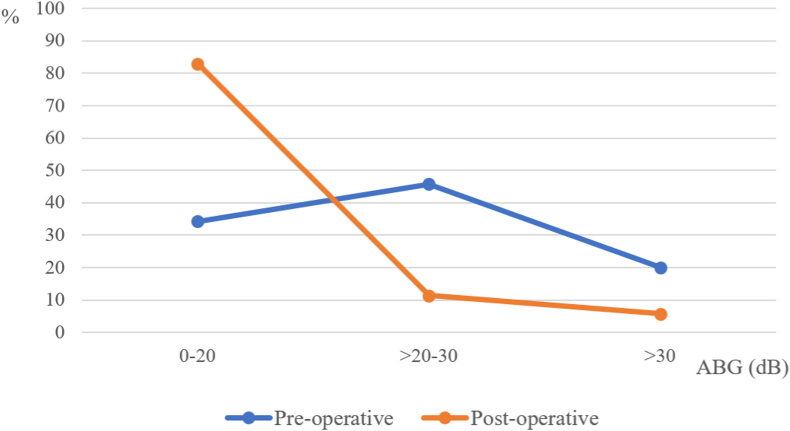
Preoperative and postoperative ABG

The mean postoperative ABG was 13.97 ± 10.03 dB, compared to the preoperative was 24.11 ± 10.79 dB. There was a significant decrease in the mean ABG (10.14 ± 8.71 dB). For successful cases of anterior tab flap underlay technique; comparison between pre- and postoperation ABG was performed by using postoperation ABG within 20 dB as the criterion. ABG of 20 dB or more before surgery was observed in 65.7% (23 patients) while 82.9% (29 patients) had their ABG within 20 dB after surgery (Fig. 2). The hearing was statistically improved with successful myringoplasty (p < 0.05).

## 4 Discussion

Classically, TM perforations have been repaired through using a variety of graft materials as well as the described techniques. According to the literature, the TM perforation closure rate was a wide range from 75 to 98% [16]. The most commonly performed technique was the underlay tympanoplasty because it was easy to operate and had a high success rate. In this technique, the graft was placed medially to the malleus and the annulus. The otologist surgeons preferred to perform the technique in posterior perforations. However, the underlay technique showed a significant disadvantage in the anterior perforation repair. Due to absence of anterior support, the applied underlay had a high risk of collapse of the anterior portion and the consequence was the failure of perforation closure as well as partial destruction of the middle ear cavity [18]. Furthermore, the access difficulty, the low level vascularization also contributed to the challenge for the success rate of the closure of the anterior TM perforation. Although the overlay technique had a high success rate for the repair of the anterior perforation, it was more technically challenging and the significant risk of graft lateralization [13].

To avoid such potential disadvantages, as well as to improve the success rate of the anterior perforations, surgeons have developed some of the modified graft techniques. In this study, we preferred to apply the surgical method developed by Primrose and Kerr for the anterior perforations. We used the endoscopy transcanal approach because of its advantages such as enhanced optics with high amplification and swift transition between the positions, visualization of the concealed areas of the middle ear and proposal a useful tool of image for the residency training. However, endoscopy transcanal surgery was a one-hand technique. So, the extensive hemorrhage occurring during elevation of the tympanomeatal flap may lead to embarrassment in the endoscopic approach particularly for inexperienced surgeons.

The age of the patients in this study ranged from 19 to 64 years, and their mean age was 35.14 ± 11.9 years. This result can be compared with several studies [20]. Our hypothesis for this is that the patients have become more cautious because of hearing impairment at this age. In our study of 35 patients, 23 patients were female and 12 patients were male with female to male ratio as 1.92:1. This could be due to the length of the eustachian tube being shorter in females compared to males [2]. All ears with perforation were divided into three groups in our study: (I) small sized perforation (1–32 mm^2^, <50%), (II) medium sized perforation (>32–48 mm^2^, 50%–75%), and (III) large sized perforation (>48 mm^2^, >75%). The number of ears in group I and III was dominant (n = 18, 51.4% and n = 12, 34.3%, respectively). As shown in Table, we noticed that the size of the perforation had an impact on the severity of hearing loss (p < 0.05). This implied that the larger the perforation of TM the greater the hearing loss. Similar results were gained in the previous studies [1,3]. To explain this result, we have mentioned two reasons. First, the consequence of the perforation was loss of effective area of the TM. Especially, with the large perforation and involving the manubrium, the lever action of the TM was deficit. This resulted in the 30 dB loss. Second, the phase differential between round window and oval window was either reduce or loss and it especially occurred at the larger perforation that these windows were exposed.

In the current study, we performed the anterior flap technique under endoscopy and our study was compared to the previous studies that used microscopy to repair the perforation with the same technique [14,15]. To secure the anterior graft and prevent the graft retraction, the anterior tab of graft was pulled into the tunnel that was created by dissection between the overlying skin and the anterior bony canal. The graft success rate (GSR) was 93%, 89.8% in the reports of the prospective study of D'Eredita and Lens in 59 individuals sample, the retrospective study of Faramarzi et al. in the sample of 157 patients, respectively. Also, we achieved an acceptable GSR (88.6%) that was in line with the previous studies. The results of our study indicated that the GSR with the size <50% was 100% while the GSR with the size >50% was 8/12 (66.7%). Similarly, the success rate of the large perforation was also reported in the prospective studies of Saleh et al. in 52 patients, Jurado et al. (71.4%, 54.54%, respectively). This was probably explained by the larger bed of the small and moderate perforations and the graft had a better chance to be taken [20]. The hearing restoration was a critical criterion for the rehabilitation in patients who suffered from TM perforation. Therefore, the otologic surgeons were more interested in improving the hearing capacity while treating the pathologies in the middle ear and mastoid cavity. The aim of the myringoplasty was closure of TM perforation and to obtain audiometric gain. The current study used the pure-tone audiometry for the assessment of hearing gain after endoscopic transcanal myringoplasty at 500, 1000, 2000 and 3000 Hz, which represent fundamental frequencies for understanding speech. The mean restoration of the air conduction hearing in our study was 10,82 ± 5,99 dB, mean ABG reduction was 10.14 ± 8.71 dB, and the percentage of ABG <20 dB increased from 34.3% to 82,9%. This result was similar to the previous studies [4,5]. On the other hand, the others reported less hearing threshold gain [19,21]. Many factors can affect the degree of hearing improvement to explain the difference, such as the status of the ossicular chain, pneumatization of the mastoid air cell, the function of the Eustachian tube, surgical technique and surgeon experiences.

### 4.1 Strengths and limitations

The strength of this study was that all patients were operated by a single surgeon. Therefore, the surgical skill has not been a confounding factor. The major limitations of this study was the lack of a matched control group and the short follow-up time. Further studies would be performed on progress to prove the effectiveness of this method.

## 5 Conclusion

To sum up, we recommended the endoscopic transcanal myringoplasty with anterior tab flap underlay technique as a suitable alternative method in anterior tympanic membrane perforation. This complementary graft technique was a solution to eliminate the disadvantages of underlay myringoplasty in repairing anterior perforations. Based on the terms of high graft success rate and hearing improvement, this technique provided a favorable outcome, even in difficult cases.

## Ethical approval

All study protocols were approved by our research ethics committee (decision number H2018/031) on May 10, 2018.

## Funding

This research was supported by science and technology project 2020 funded by 10.13039/501100017575Hue University (DHH2020-04-135).

## Author contributions

Thanh Thai Le, Doan Minh Nhat Vo, Thi My Duong, Nguyen Nguyen: Data collection, Manuscript writing, Results discussion.

Nguyen Nguyen, Thanh Thai Le, Doan Minh Nhat Vo: Manuscript writing and revision.

Thanh Thai Le: Paper revision.

## Registration of research studies

1. Name of the registry: N/a
2. Unique Identifying number or registration ID: N/a
3. Hyperlink to your specific registration (must be publicly accessible and will be checked): N/a

## Guarantor

Nguyen Nguyen is the guarantor of the study and accept full responsibility for the work and/or the conduct of the study, had access to the data and controlled the decision to publish.

## Provenance and peer review

Not commissioned, externally peer-reviewed.

## Consent

Written informed consent was obtained from the patient for publication of this case report and accompanying images. A copy of the written consent is available for review by the Editor-in-chief of this journal on request.

## Declaration of competing interest

We have no known conflict of interest to disclose.
